# Quality of life and its association with psychiatric disorders in outpatients with trauma history in a tertiary hospital in Kathmandu, Nepal: a cross-sectional study

**DOI:** 10.1186/s12888-021-03104-6

**Published:** 2021-02-16

**Authors:** Saraswati Dhungana, Rishav Koirala, Saroj Prasad Ojha, Suraj Bahadur Thapa

**Affiliations:** 1 Department of Psychiatry and Mental Health, Institute of Medicine, Tribhuvan University, Kathmandu, Nepal; 2 Division of Mental Health and Addiction, Institute of Clinical Medicine, University of Oslo, Oslo, Norway; 3 Brain and Neuroscience Center, Kathmandu, Nepal; 4 Division of Mental Health and Addiction, Oslo University Hospital, Oslo, Norway

**Keywords:** Quality of life, Trauma, Anxiety, Depression, Post-traumatic stress disorder, Clinical setting

## Abstract

**Background:**

Quality of life is an important indicator of health and has multiple dimensions. It is adversely affected in patients with trauma history, and psychiatric disorders play an important role therein. Studies in trauma-affected populations focus mainly on the development of psychiatric disorders. Our study explored various aspects of quality of life in trauma patients in a clinical setting, mainly focusing on the association of psychiatric disorders on various domains of quality of life.

**Methods:**

One hundred patients seeking help at the psychiatry outpatient of a tertiary hospital in Kathmandu, Nepal, and with history of trauma were interviewed using the World Health Organization Composite International Diagnostic Interview version 2.1 for trauma categorization. Post-traumatic stress disorder symptoms were assessed using the Post-Traumatic Stress Disorder Checklist-Civilian Version; while the level of anxiety and depression symptoms was assessed using the 25-item Hopkins Symptom Checklist-25. Quality of life was assessed using the World Health Organization Quality Of Life-Brief Version measure. Information on sociodemographic and trauma-related variables was collected using a semi-structured interview schedule. The associations between psychiatric disorders and quality of life domains were explored using bivariate analyses followed by multiple regressions.

**Results:**

The mean scores (standard deviations) for overall quality of life and health status perception were 2.79 (.87) and 2.35 (1.11), respectively. The mean scores for the physical, psychological, social and environmental domains were 12.31 (2.96), 11.46 (2.84), 12.79 (2.89), and 13.36 (1.79), respectively. Natural disaster was the only trauma variable significantly associated with overall quality of life, but not with other domains. Anxiety, depression and post-traumatic stress disorder were all significantly associated with various quality of life domains, where anxiety had the greatest number of associations.

**Conclusion:**

Quality of life, overall and across domains, was affected in various ways based on the presence of psychiatric disorders such as anxiety, depression and post-traumatic stress disorder in patients with trauma. Our findings therefore emphasize the need to address these disorders in a systematic way to improve the patients’ quality of life.

**Supplementary Information:**

The online version contains supplementary material available at 10.1186/s12888-021-03104-6.

## Background

Trauma has been defined in the tenth revision of the International Classification of Diseases (ICD-10) as any stressful event with a probability of causing severe distress in almost anyone, for example, natural disasters such as earthquake, flood, hurricane or manmade trauma, such as torture, war, terrorism, violent accident, rape, or physical or sexual assault [1]. Trauma victims are a unique population group and react differently depending on their past experiences, personality, and several other factors such as exposure type, extent of loss, coping style and country’s socio-economic and political structure [2–5]. A number of studies have examined the short-term and long-term consequences of trauma mostly in the general population [6–8], but also in the patient population [9–11]. Studies have mostly focused on the morbidity aspects of trauma victims [4] undermining the quality of life (QOL) in this population which is an important construct to consider when discussing the impacts of trauma. Recently, progress is underway from traditional indicators of health such as mortality and morbidity to more holistic approach and considering the aspects of wellbeing and functioning [12–14]. QOL indicators have gained increased attention in the treatment packages of psychiatric diagnoses today [12].

QOL does not have a single consensus definition [15, 16]. It has varied definitions and assessment tools; however, many agree that the patients’ subjective experiences should be given importance while assessing this important construct [12, 15, 16]. The definition given by the World Health Organization (WHO) is comprehensive, taking into considerations the aspects of culture in which people live and function [13]. The WHO defines QOL as ‘individuals’ perceptions of their position in life in the context of the culture and value systems in which they live and in relation to their goals, expectations, standards and concerns’ [13].

### Quality of life and psychiatric diagnoses in trauma

Psychiatric diagnoses in trauma patients include anxiety disorders, depression, post-traumatic stress disorder (PTSD) and substance-use disorders, either singly or in some combination [16–19]. Many studies have associated PTSD diagnosis after trauma with poor QOL [16, 20–26]. In both clinical and population studies, people with anxiety disorders, regardless of the type, seem to have impaired QOL in the domains of psychological wellbeing and social relations, and the effect appears to remain relevant irrespective of sociodemographic factors, comorbidities and severity of symptoms [23]. Depression in patients with PTSD seems to have an effect on QOL across multiple domains [16, 19]. Trauma victims have impaired QOL regardless of whether they develop psychiatric disorders or not [26–28].

### Quality of life and trauma-related variables

Studies have suggested associations of QOL domains with different trauma types such as earthquake [29], war/combat [12], and sexual abuse [30]. Those with history of multiple traumas and those who perceive trauma as life-threatening are more vulnerable to having poor QOL [30].

### Psychiatric diagnoses, quality of life and sociodemographic variables

Female gender [31–35] and elderly age [30] have been identified as risk factors for PTSD development following trauma exposure. Being single, unemployed, head of the family, low income [36], history of psychiatric illness [34], and lower education level [34] conferred greater risk for PTSD development following various types of trauma such as war [37] and earthquake [32]. Some studies [25, 30] have also explored QOL, while others did not. Wen et al. found that higher education and higher income were correlated positively, while age correlated negatively with QOL [22]. A meta-analysis of PTSD risk factors however concluded that the effect of gender was significant only in studies with civilian samples, while young age at trauma and lack of education were significant in military samples. The only variable with consistent and homogeneous effects as a predictor of PTSD in both samples was a family history of psychiatric illness [37], demonstrating the complex relationship of many factors in predicting PTSD. One study explored the determinants of PTSD and depression after a major hurricane, where the sociodemographic factors of low household income and lower education status were associated with depression and not PTSD [38]. A study of Cambodian civilians in the four decades following the genocide found that higher age was associated with depression and anxiety; female gender was associated with higher reporting of anxiety, depression and PTSD; urban dwelling residents reported more PTSD but no anxiety or depression; low education was associated with increased rates of depression and anxiety; and having no savings was associated with increased rates of anxiety, depression and PTSD [39].

QOL as a construct has a complex relationship with multiple factors such as sociodemographic, trauma-related, and clinical diagnoses after trauma exposure. Nepal, a country in South Asia, is quite vulnerable to traumas of all kinds because of its unstable geopolitical structure [40]. A handful of small-scale studies on clinical diagnoses after trauma have been conducted in the general population [41, 42] and in clinical settings [43]. To our knowledge, no study has been conducted on QOL in trauma victims, in the clinical population in the Nepali setting and in South Asia. We therefore wanted to explore multiple QOL domains in the patient population with history of trauma. Our research aim was to determine if psychiatric diagnoses such as anxiety, depression and PTSD after trauma exposure in this patient population were associated with poor QOL in various domains.

## Methods

### Participants

This study is a part of a large-scale study, i.e. ‘Study of health outcomes after trauma’ (SHOT) [43, 44], where we attempted to measure the QOL of patients with trauma history. For the operational definition of trauma, we used the WHO Composite International Diagnostic Interview (CIDI) version 2.1 section K, which has a list of 10 traumatic events that qualify as trauma in our study participants. This was a naturalistic cross-sectional study. Participants were recruited from the outpatient department (OPD) of the Department of Psychiatry and Mental Health of Tribhuvan University Teaching Hospital, Kathmandu, Nepal. Those coming to the OPD, and with a positive history of trauma were invited to participate in the study if they met the inclusion criteria.

#### Inclusion and exclusion criteria

The inclusion criteria were age between 18 and 60 years and history of at least one trauma at least 1 month prior to the study period as defined by the ICD-10 PTSD section. Patients were excluded if they had serious medical or neurological illness, organic mental disorder, psychotic disorders, history of severe head trauma, cerebral infection and dyslexia, which was determined via inquiring with the patients and checking their past medical history.

Sample size:

The sample size was calculated using the following formula:

$$ \mathrm{n}={\left({z}_1-\alpha /2\right)}^{2\ast}\kern0.5em \left(\mathrm{p}\right)\left(\mathrm{q}\right)/{\left(\mathrm{d}\right)}^2, $$

where

n = desired sample size,

(z_1−_ α _/2_)^2^ = 1.96 for 95% confidence interval,

p = prevalence of trauma-related disorders based on previous research [42], which was 50%,

$$ \mathrm{q}=1-\mathrm{p}, $$

d = 10%, i.e. precision of estimate.

This formula yielded a required sample size (n) = 96.04 ~ 96.

Considering a possible 10% dropout from the study, the total required sample size was 106. A total of 102 patients were recruited, but because two did not meet the criterion for age, they were excluded, and we had a total number of 100 patients instead. For one patient, data on many variables were missing so we included 99 patients for our analyses purpose. The data were collected over a period of 16 months from 1 April, 2017 to 14 August, 2018.

### Procedure and tools

Patients who agreed to participate were provided detailed information regarding the project and the aims of the study. Written informed consent was obtained from each participant prior to conducting the interviews. For illiterate participants, the accompanying informant provided the consent on their behalf after detailing about the consent process. Information on the study and the possibility of withdrawing voluntarily was given both in written form and verbally.

#### Ethical issues

Ethical clearance was obtained from the Nepal Health Research Council (NHRC, reference number 801) and the Institute of Medicine Institutional Review Committee (IRC, reference number 480 (611)6^2^/ 075/076), Nepal, and the Research Ethical Committee (REK), Norway (reference number 2015/2081). The methodology has been detailed previously [43].

A semi-structured questionnaire was designed specifically for the participants to obtain sociodemographic variables such as age, gender, place of residence, marital status, education status, socioeconomic status, employment level and trauma-related variables such as trauma type and number of traumas experienced. Place of residence was categorized into two groups: rural and urban, depending on where the participant resided at the time of trauma. Marital status was categorized into two broad groups: married or single (including unmarried, divorced or widowed). Education status was categorized into three groups: illiterate, up to high school, and above high school. Religion was broadly categorized into two groups: Hindu, and others, as the majority of the Nepali population are Hindu by religion. The socioeconomic status (SES) of the participants was assessed using the Kuppuswami SES scale [45]. SES was categorized into five classes: upper, upper middle, lower middle, upper lower and lower. Trauma type was broadly categorized into two groups: natural and others (i.e. torture/terrorism, accident, kidnap/threatened with weapon, witness murder/rape/grievous injury, rape, sexual assault (other than rape), physical assault, close one suffering from stressful event, war, and any other extremely stressful events). Whenever a participant reported past experience of both types of trauma, we recorded only the trauma considered by the participant to be the most distressing. As the median number of lifetime trauma exposure of our participants was two, we grouped trauma numbers in two categories: up to two traumas, and > 2 traumas. The interview time varied between participants depending on the type and number of traumas and also based on the presence or absence of psychiatric diagnoses. The average interview time per participant was 2 h.

##### WHO World Mental Health Composite International Diagnostic Interview (WHO WMH-CIDI) version 2.1

The WHO WMH-CIDI is a fully structured interview for diagnosing current and lifetime psychiatric disorders as per the criteria set by ICD-10 and the fourth edition of the Diagnostic Statistical Manual of Mental Disorders (DSM-IV) and was first developed in 1980 by the WHO. It is available in computer and paper and pencil form, where the paper and pencil form is mostly used in epidemiological studies. The psychometric properties of the CIDI, including its reliability and validity, are acceptable to good [46]. CIDI version 2.1 has been validated in the Nepali language [47]. Section K contains a list of 10 traumatic events that served as a reference to qualify trauma in the participants.

##### The PTSD symptom checklist-civilian version (PCL-C)

We used the PCL-C to measure current PTSD symptoms and diagnosis. This is a 17-item self-rated scale that parallels the diagnostic criteria B, C, and D for PTSD as described in the DSM-IV [48]. Items are rated on a Likert scale from 1 (not at all) to 5 (extremely) to indicate the degree of subjective symptoms over the past month. Different studies have shown that the PCL-C has good psychometric properties, including internal consistency, test–retest reliability, convergent validity, and discriminant validity [48–50]. Its psychometric properties have also been tested in Nepal [47]. We used the PCL-C scores as measures of PTSD in all our analyses.

##### Hopkins symptom Checklist-25 (HSCL-25)

This is a Likert type scale with a total of 25 items derived from the 90-item symptom checklist (SCL) [51]. Part I has 10 items for anxiety while part II has 15 items for depression. Each item is rated on a scale of 1 (not at all) to 4 (extremely) for symptoms within the last 2 weeks, and two scores are calculated. The total score is the average of all 25 items, while the anxiety and depression scores are the averages of the 10 and the 15 corresponding items, respectively. The scale has been widely used in different populations across nations and has good psychometric properties [52]. In Nepal, it has been validated against depression using the CIDI [47]. In the present study, HSCL-25 individual scores were used for diagnosing anxiety and depression.

##### WHO Quality of Life-Brief Version (WHOQOL-BREF)

The WHOQOL-BREF was derived from the complete WHOQOL 100-item version. The complete version was developed in around 15 cultural settings and tested in 37 field trials over several years. It is now available in 29 languages. It provides scores across multiple domains and therefore is a comprehensive measure of QOL. The shorter WHOQOL-BREF, with 26 items and good psychometric properties, was later developed and used very frequently in research across nations and settings because of its brevity and simplicity [13]. The first two questions assess overall perception of QOL, and perception of health, respectively, while other questions have been transformed into four domains: physical, psychological, social, and environmental. The physical domain examines the person’s physical discomfort and the sensation of control or relief over pain due to any measures. It also covers the person’s fatigue and enthusiasm in pursuing day to day goals, taking into consideration the effect of sleep and rest. The psychological domain focusses on the person’s mental makeup and their subjective perception in terms of self-esteem, integrity and control over their life. The social domain examines social connections as well as the support system, while the environmental domain covers aspects of housing, safety and security, access to health and other opportunities in terms of affordability and quality and how that impacts QOL [13]. The domain scores are calculated by computing the means of items within each domain, and higher scores indicate better QOL, as described in the manual. Here, we used the Nepali version of the WHOQOL-BREF used by Giri et al. [53].

### Statistics

Means and standard deviations (SDs) for continuous variables, and frequencies for categorical variables were used as descriptive statistics. The normal distribution of the continuous variables was checked by using skewness and kurtosis ±3.29 [54], and as they were normally distributed, independent sample t tests and analysis of variance (ANOVA) tests were used to determine if there were significant differences between the means for QOL scores for trauma type, trauma number, and sociodemographic variables. Data are expressed as mean (SD), and percentage, wherever appropriate.

Bivariate analyses were first performed between the different dependent and predictor variables to examine their associations using Spearman’s rank correlation and Pearson’s correlation where applicable. The first two items of QOL were treated as ordinal items, while gender was coded as a binary item, with female = 1 and male = 0. In the final models, ordinal linear regression was used via a generalized linear model for the first two items of QOL domains, where these two QOL indicators were retained as dependent variables, while the mean PTSD and mean anxiety and depression scores measured from PCL-C and HSCL-25, respectively, along with age and gender, were retained as predictor variables. In another final model, multivariate regressions were performed to predict the four QOL domains, i.e. physical, psychological, social and environmental; again, the mean PTSD scores (PCL-C) and mean anxiety and depression scores (HSCL-25) along with age and gender were retained as predictor variables. Preliminary analyses were conducted to ensure no major violation of normality, linearity, multicollinearity and homoscedasticity. A *p*-value of .05 was taken as significant in all statistical tests. Data were analysed using Statistical Package for the Social Sciences (SPSS) 26 (IBM SPSS Statistics for Windows, Armonk, NY: IBM Corp.).

## Results

The mean participant age was 33.31 (10.44) years. An almost equal number of males (48%) and females (52%) were represented in the study. Seventy-nine percent of the participants were married, while the remaining 21% were single, separated, divorced or widowed. The vast majority (82%) practiced Hinduism, while the rest belonged to other religious backgrounds such as Christianity, Islam or Buddhism. More than half (56%) had completed high school, while 27% had completed more than high school, and 17% were illiterate. Forty-six percent of the participants had upper middle SES, 34% had lower middle SES, 12% had upper SES and 8% had lower SES. An almost equal number of participants came from rural (49%) and urban (51%) backgrounds. The majority (78%) of the participants suffered less or equal to at least two major traumas, while the rest (22%) had experienced > 2 traumas. Based on trauma type, slightly more than half (57%) had experienced natural disaster, while 43% experienced other traumas.

### Quality of life mean scores

The mean scores on overall perception of QOL and overall perception of health are presented in Table 1, and the mean scores on the four QOL domains are presented in Table 2. The means (SD) for overall perception of QOL and perception of health were based on single-item measure of the first two items of the WHOQOL-BREF measured on a 5-point rating scale.

**Table 1:** Means, Standard Deviations, and Intercorrelations for predictor variables and perception of overall quality of life and perception of satisfaction with health scales

*N* = 99.									
Variables	M	SD	1	2	3	4	5	6	7
1. Overall quality of life+	2.79	0.87	–						
2. Overall satisfaction with health+	2.35	1.11	0.36**	–					
3. Anxiety mean score from HSCL	2.11	0.64	−0.48**	−0.48**	–				
4. Depression mean score from HSCL	2.19	0.69	−0.34**	−0.53**	0.63**	–			
5. PTSD mean score from PCL-C	2.14	0.81	−0.48**	−0.40**	0.68**	0.57**	–		
6. Age	33.31	10.44	0.003	−0.13	−0.13	0.03	−0.07	–	
7. Gender++	0.52	0.50	−0.28	0.04	0.16	0.03	0.13	0.12	–

*M* mean, *SD* standard deviation, *N* number of participants, *HSCL* Hopkin’s symptom checklist, *PTSD* post-traumatic stress disorder, *PCL-C* PTSD checklist civilian version

+ = Ordinal items, ++ = Binary item (Female =1, Male = 0)

*p < .05. **p < .01

**Table 2:** Means, Standard Deviations, and Intercorrelations for predictor variables and domains of quality of life scales

*N* = 99.											
Variables	M	SD	1	2	3	4	5	6	7	8	9
1. Physical domain	12.31	2.96	__								
2. Psychological domain	11.46	2.84	.61**	__							
3. Social domain	12.79	2.89	.39**	.60**	__						
4. Environmental domain	13.36	1.79	.34**	.47**	.37**	__					
5. Anxiety mean score from HSCL	2.19	.69	−.59**	−.53**	−.20*	−.39**	__				
6. Depression mean score from HSCL	2.11	.64	−.64**	−.77**	−.59**	−.44**	.66**	__			
7. PTSD mean score from PCL-C	2.14	.81	−.52**	−.63**	−.37**	−.38**	.57**	.68**	__		
8. Age	33.31	10.44	−.15	.03	−.03	.01	−.03	.01	−.07	__	
9. Gender	.52	.50	−.05	.00	.00	.00	.03	.16	.13	.12	__

*M* mean, *SD* standard deviation, *N* number of participants, *HSCL* Hopkin’s symptom checklist, *PTSD* post-traumatic stress disorder, *PCL-C* PTSD checklist civilian version

*p < .05. **p < .01

### Bivariate relations between psychiatric diagnoses and quality of life

Bivariate analyses suggested significant association of overall perception of QOL; overall perception of health; and the physical, psychological, social and environmental QOL domains with psychiatric diagnoses such as anxiety and depression as assessed by the HSCL-25 and PTSD as assessed by the PCL-C. Age and gender had no significant associations with the QOL scales (Tables 1 and 2).

### Factors associated with quality of life

Although they were not statistically significant in the bivariate analysis, age and gender were also retained in the final regression models because various studies identified them as important demographic predictors [30, 39, 55]. Here, the regression models identified anxiety, depression, and PTSD as significant predictors when multivariate regression analysis was run for overall QOL, health status perception, and all four QOL domains. On individual prediction, anxiety was the single most significant predictor of overall perception of health, physical domain, psychological and social domains. Depression was independently significant for predicting overall perception of QOL status, and the physical and social domains. PTSD was an independent predictor of overall perception of QOL status and the psychological domain. None of the independent variables was a predictor of the environmental domain (Tables 3 and 4).

**Table 3:** Ordinal linear regressions (Generalized linear models) for overall perception of life and perception of health as dependent variables and mean anxiety, mean depression and mean PTSD scores, age and gender as predictor variables

	Overall perception of QOL			Perception of health		
	OR	95% CI LB UB	*P* value	OR	95% CI LB UB	*P* value
Variables (*N* = 99)						
Anxiety	.91	.42 1.98	.81	.58	.41 .83	.003**
Depression	.32	.12 .84	.02*	.67	.43 1.03	.07
PTSD	.46	.23 .94	.03*	.89	.65 1.22	.47
Age	1.00	.96 1.04	.95	.99	.98 1.01	.55
Gender	1.34	.62 2.92	.46	1.20	.83 1.73	.33

Ordinal regression models in SPSS was used to get the parallel lines which is like linearity assumption for linear models

*QOL* Quality of life, *Q1* Item 1 of QOL, *Q2* Item 2 of QOL, *OR* odds ratio, *CI* confidence interval, *LB* lower bound, *UB* upper bound, *N* number of participants, *PTSD* posttraumatic stress disorder

*p < .05. **p < .01

**Table 4:** Multivariate linear regressions for four domains of quality of life as dependent variables and mean anxiety, mean depression, mean PTSD scores and age and gender as predictor variables

Variables (*N* = 99)	Physical domain			Psychological domain			Social domain			Environmental domain		
	β	95% CI LB UB		β	95% CI LB UB		β	95% CI LB UB		β	95% CI LB UB	
Anxiety	−1.81**	−2.87	−.76	−2.92***	−3.78	−2.07	−3.68***	−4.78	−2.59	−.76	−1.54	.03
Depression	−1.17**	− 2.04	−.29	.08	−.63	.79	1.48**	−5.8	2.39	−.41	−1.06	.24
PTSD	−.40	−1.16	.35	−.76*	−1.38	−.14	−.14	−.93	.65	−.24	−.81	.32
Age	−.04	−.08	.00	.009	−.02	.04	−.009	−.05	.03	.003	−.03	.03
Gender	.04	.93	−.85	.71	−.02	1.43	.69	−.24	1.61	.20	−.46	.86
F value	17.88			31.83			13.42			5.28		
R square	.49			.63			.42			.22		

*PTSD* posttraumatic stress disorder, *N* number of participants, *β* unstandardized regression coefficient, *CI* confidence interval, *LB* lower bound, *UB* upper bound

*p < .05. **p < .01. ***p < .001

### Trauma-related variables and quality of life

Regressions were done for different trauma types, where trauma was dichotomized as natural disaster and others, and also on the numbers of traumas experienced by the participants, i.e. < 2 and > 2, and adjusting for age and gender. The models were not significant for predicting any domain or overall perception of QOL and health, although the natural disaster trauma type was an independent predictor for overall perception of QOL (R^2^ = .05, F = 1.16, *p* = .04 and β = −.39).

### Sociodemographic variables and quality of life

Sociodemographic variables such as age, gender, education of the head of the family, occupation of the head of the family, family income, SES, marital status, and religion were entered into regression in a separate model to determine if they predicted QOL not taking into consideration the psychiatric diagnoses. None of the sociodemographic variables seemed to affect QOL in the standard regression models.

## Discussion

In our study, highest mean score of 13.36 (1.79) was recorded for the environmental domain and lowest mean score was for the psychological domain 11.46 (2.84). Similar findings have been reported in a study from Norway, where the environmental domain had the highest score, but contrary to our findings, the physical domain had the lowest score [56]. Psychological wellbeing constitutes many facets of a person, such as perceived body image, self-esteem and self-confidence, and emotions such as thinking and feeling. Patients with most psychiatric disorders seem to have major disturbance in thinking and feeling, with disturbed self-image, and have low self-esteem because of the prevailing stigma against mental illness [57], and this provides an explanation of the lowest mean scores for this particular domain compared to that of the others. On the other hand, the social domain constitutes interpersonal relations and sexual activity with an element of social support, while the environmental domain covers aspects such as safety and security, home environment, finances, opportunities for recreation, and transport [13], and these domains might have scored better because of the strong family values and social networks in Nepali culture.

Our findings reveal that psychiatric diagnoses are associated with different aspects of QOL. Other studies have reported similar finding in those exposed to trauma [20, 56, 58]. In our study, anxiety was the single most important predictor, as it was associated with overall perception of health, and the physical, psychological, and social domains. A meta-analysis of patients with anxiety disorders with control samples from a non-clinical population reported similar findings, but here, only two domains, i.e. psychological and social, were affected. This could be because trauma history was not considered, and PTSD was also included within the anxiety disorders and was not categorized separately [23].

In our study, depression was significantly associated with overall perception of QOL, and the physical and social domains. However, this was not consistent with other studies. Araujo et al. found that depression affected all QOL domains [16]. Another study also showed a modest association between depression and physical health and mental wellbeing [59]. Again, there could be a number of explanations, as the measures of depression and scales for assessing QOL differed, as did the methodologies of the previous studies. Even if the methodologies were similar and the authors had used similar instruments for the assessment, this could be because of the tendency of mental health professionals to make one diagnosis at a time. The ICD-10, the standard classification system for mental disorders, enables hierarchical diagnosis. Many clinicians thus do not go on to further diagnose other psychiatric diagnoses, leading to under-diagnosis of anxiety and PTSD. Another explanation could be the high frequency of comorbidities seen in these disorders after any trauma. Depression and PTSD are often comorbid [16], and many symptomatology could fit into either of the diagnoses leading to underreporting of one diagnosis over the other depending on what the study focuses on.

In our study, PTSD scores predicted overall perception of QOL and psychological wellbeing in the final regression models. Similar findings were reported in a study from Norway, where both depression and PTSD were associated with reduced overall QOL even 6 years after the trauma [56]. A study conducted among specialized clinic patients and another study among American veterans found that PTSD was associated with all the domains [30, 60].

Another study found that PTSD negatively affected the physical and psychological domains [16]. PTSD symptoms were significantly associated with the mental wellbeing domain only in pension-seeking Canadian veterans [59] and with the social domain only in a study involving firefighters [61]. Therefore, although all studies concluded on the negative association between PTSD and QOL, they differed on the QOL domains affected by having PTSD. A few important observations here could explain this. First, the studies all used different methodologies; second, the populations and settings were differed, ranging from pension-seeking veterans to help-seeking clinical population; third, the outcome measures for QOL were different, with some focusing only on the physical and psychological aspects while some focused on emotional aspect only. Fourth, the PTSD assessment measures were different, and also in most studies, only PTSD was assessed rather than other major psychiatric disorders. To our knowledge, studies on QOL across psychiatric disorders after traumatic experiences are very scarce in South Asia and in the Nepalese context in particular. The only study from Asia we found was from Taiwan following a major gas explosion in 2014; the study reported an association between PTSD and depression and various aspects of QOL, although the study population was not a clinical sample [62]. We came across a few studies from Asia linking QOL with psychiatric disorders in specific populations rather than trauma victims. Depression severity was associated with poor QOL in outpatients from Singapore [63]. Studies among the elderly from Korea and Bangladesh also reported associations between depression and lower QOL [64, 65]. The only study from Nepal assessing QOL after major trauma was also among the elderly from a city area, where higher age and PTSD diagnosis were associated with poorer QOL even 1.5 years after the trauma [66]. Another study from Nepal on old-age home residents also reported associations between depression and all four QOL domains [67]. Therefore, the limited studies available show that psychiatric conditions such as PTSD and depression are associated with poorer QOL. However, there are several points to consider before the findings can be generalized. Very few studies we obtained were performed among specific populations or in those where history of trauma was not considered. Further, only one psychiatric condition was considered, such as depression or PTSD, rather than all three as included in our study.

In the present study, there were two trauma variables, namely trauma type (i.e. natural or others) and trauma number. We grouped all other traumas besides natural disaster as others, as they were much fewer in number. In our sample, trauma was not significantly associated with QOL domains except for natural trauma, which was associated with overall QOL. Natural disasters such as earthquakes, floods and tsunamis are uncertain and are of such large scale that they disrupt families, societies and living places, and at the same time make people feel that life is unpredictable, leading to disruption, and overall impairment of QOL, which could explain the results we obtained. Another possible reason is the nature of social support people receive during times of natural traumas, as the majority of the population gets involved.

This finding is quite different from that of other studies, where trauma was associated with QOL in studies involving refugee status [30], sexual assault [30], rape and war/combat [60].

Our findings could be explained with a number of reasons. First, trauma is a broad term encompassing various types. In our study, trauma type was categorized as either natural or others. Ten different types of trauma were included in the others category as listed in section K of the CIDI version 2.1. Second, several participants reported more than one type of trauma exposure, but we recorded only the trauma considered most distressing by the participant and included that in the analyses of QOL. This is also in accordance with the classification by the CIDI, where major trauma considered the most distressing by the participant is classified as the primary trauma. Besides, trauma severity and extent rather than number, personality of a person, past psychiatric history and social support all play more vital roles [37].

In the present study, sociodemographic variables were not significantly associated with QOL. Most studies have suggested otherwise. One study reported positive correlations between higher education and higher income, while, age correlated negatively with QOL [22].

Female gender [33, 68], old age [30], lower income [36] and low education level [34] were identified as risk factors for PTSD following trauma. As PTSD and depression affect QOL negatively, sociodemographic variables associated with higher risk of leading to PTSD or depression might be inferred. However, direct correlation with QOL is difficult. Rather than the sociodemographic characteristics, studies have suggested that many factors combine in the development of a psychiatric diagnosis in patients with trauma exposure, such as history of childhood abuse [17, 69], history of psychiatric illness, personality [69], coping, severity and extent of trauma, perception of trauma threat and social support [69].

### Limitations

Our study is the first of its kind in Nepal and in South Asia to explore the aspects of QOL in the clinical population with trauma history at a tertiary hospital. We used objective rating scales to assess the level of anxiety, depression and PTSD symptoms, while sociodemographic and trauma-related information was collected using a semi-structured proforma designed for this study. All interviews were carried out by a qualified psychiatrist and there was limited missing information during data analysis. However, the sample size was small, and it was a clinical population from a single centre, so the generalizability of the results to non-clinical population is limited. We also used convenience sampling. Although we calculated the sample size using a standard formula, some of the findings might be due to inadequate power. The major limitation was that age and gender, as demographic variables, were included in the final regression models for obtaining results although they were not statistically significant in the bivariate analysis. Further, the majority of the participants reported experiencing > 1 trauma, and some reported having been exposed to both natural and others trauma; however, this was not taken into consideration during the analyses, so correlating trauma types with QOL was difficult. Although trauma was an important variable, we did not control for trauma in our final regressions. Another major limitation is that this was a cross- sectional study, so the directionality of the clinical diagnoses and QOL domains is questionable. We also do not have information on the predictability of the clinical diagnoses on the QOL domains. There was no comparison group, meaning we had no patients with trauma and without diagnoses of any psychiatric disorders under study and the study of QOL in this particular group of patients could have yielded a better conclusion regarding the association of psychiatric disorders with QOL.

## Conclusions

QOL across multiple domains is affected in patients with trauma history, and these aspects are variably affected by anxiety, depression and PTSD, not considering their sociodemographic and trauma-related variables. More than PTSD, anxiety and depression seem to exert greater effects on various aspects of QOL. Assessing QOL is therefore important when examining patients with clinical diagnoses of anxiety, depression and PTSD in patients with trauma and addressing these disorders post-trauma has a huge impact on improving their QOL. We recommend further studies on the QOL of this population, with more critical research design over larger samples, across settings, and with longitudinal design.

## Supplementary Information

**Additional file 1.**

## Data Availability

The datasets used and/or analysed during the current study are included in this published article (and its supplementary information files).

## Abbreviations

ICD-10: International Classification of Diseases, tenth revision
QOL: Quality of Life
WHO: World Health Organization
PTSD: Post-traumatic stress disorder
SHOT: Study of health outcomes after trauma
OPD: Outpatient Department
NHRC: Nepal Health Research Council
IRC: Institutional Review Committee
REK: Research Ethics Committee
WHO WMH-CIDI: WHO World Mental Health Composite International Diagnostic Interview
DSM-IV: Diagnostic Statistical Manual, fourth edition
PCL-C: PTSD Symptom Checklist-Civilian version
HSCL-25: Hopkins Symptom Checklist-25
SCL: Symptom Checklist
WHOQOL-BREF: WHOQOL-Brief Version
SD: Standard deviation
ANOVA: Analysis of variance
SPSS: Statistical Package for the Social Sciences
